# Impact of dehydration on perceived exertion during endurance exercise: A systematic review with meta-analysis

**DOI:** 10.1016/j.jesf.2022.03.006

**Published:** 2022-04-13

**Authors:** Thomas A. Deshayes, Timothée Pancrate, Eric D.B. Goulet

**Affiliations:** a Faculty of Physical Activity Sciences, University of Sherbrooke, P.Q., Canada; b Research Center on Aging, University of Sherbrooke, P.Q., Canada

**Keywords:** Hydration, Hypohydration, Performance, Rating of perceived exertion, RPE

## Abstract

**Background:**

Understanding the impact of stressors on the rating of perceived exertion (RPE) is relevant from a performance and exercise adherence/participation standpoint. Athletes and recreationally active individuals dehydrate during exercise. No attempt has been made to systematically determine the impact of exercise-induced dehydration (EID) on RPE.

**Objective:**

The present meta-analysis aimed to determine the effect of EID on RPE during endurance exercise and examine the moderating effect of potential confounders.

**Data analyses:**

Performed on raw RPE values using random-effects models weighted mean effect summaries and meta-regressions with robust standard errors, and with a practical meaningful effect set at 1 point difference between euhydration (EUH) and EID. Only controlled crossover studies measuring RPE with a Borg scale in healthy adults performing ≥30 min of continuous endurance exercise while dehydrating or drinking to maintain EUH were included.

**Results:**

Sixteen studies were included, representing 147 individuals. Mean body mass loss with EUH was 0.5 ± 0.4%, compared to 2.3 ± 0.5% with EID (range 1.7–3.1%). Within an EID of 0.5–3% body mass, a maximum difference in RPE of 0.81 points (95% CI: 0.36–1.27) was observed between conditions. A meta-regression revealed that RPE increases by 0.21 points for each 1% increase in EID (95% CI: 0.12–0.31). Humidity, ambient temperature and aerobic capacity did not alter the relationship between EID and RPE.

**Conclusion:**

Therefore, the effect of EID on RPE is unlikely to be practically meaningful until a body mass loss of at least 3%.

## 1 Introduction

Rating of perceived exertion (RPE), a subjective estimation of the intensity or difficulty of a physical task, is widely used by professionals in the field of exercise sciences, coaching and sports medicine to monitor or prescribe exercise intensity.^1^ Developed by Gunnar Borg,^2^^,^^3^ the Borg RPE scale is a universally accessible, comprehensible, useful, non-invasive, valid and inexpensive tool that can be used in diverse populations such as in children, adolescents, young and older adults, and under different conditions, including leisure and elite sports, clinical rehabilitation and scientific research. Although the etiology of RPE is unclear, it is proposed that it is either centrally derived^4^ or generated by neuronal processes that integrate afferent signals from various peripheral and central sources, as well as from psychological factors.^3^^,^^5^

The RPE is a pivotal component of aerobic exercise. Indeed, it acts as a regulator of exercise intensity^6^^,^^7^ and exercise duration^8^, ^9^, ^10^ and, thus, modulates exercise capacity in competitive athletes and, as important, is at the core of the decision to engage and adhere to the regular practice of physical activity among recreationally active individuals.^11^^,^^12^ Given that physiological and psychological signals can act individually or in concert to disturb RPE, it follows that limiting the number of physiological or emotional stressors to a minimum during exercise should ensure optimal performance for the athlete and lead to a sense of fulfillment in recreationally active individuals.^6^

Depending on a host of factors, sweat losses typically reach 0.5–1.7 L/h during exercise.^13^ Athletes as well as recreationally active individuals do not usually replace all their fluid losses during exercise. Exercise-induced dehydration (EID), best represented by the acute body mass loss accrued during exercise, alters thermoregulatory, metabolic and cardiovascular functions,^14^, ^15^, ^16^, ^17^, ^18^ more particularly in individuals with low aerobic fitness^19^ and may predispose to the development of thirst, headaches, tiredness, mental fatigue^20^ and impaired mood,^21^^,^^22^ while its impact upon cognitive performance is still debated.^23^ These factors may contribute to increasing RPE during exercise, ultimately impeding exercise performance in athletes^24^ and potentially decreasing exercise adherence and participation in recreationally active individuals^12^^,^^25^ which, from a societal and health perspective, is not suitable. Indeed, both the affective response and RPE are associated with long-term physical activity participation.^26^

The relationship between EID and RPE has received much scientific attention, with some studies showing that EID can significantly increase RPE,^15^, ^16^, ^17^, ^18^^,^^27^, ^28^, ^29^, ^30^, ^31^, ^32^, ^33^, ^34^, ^35^, ^36^, ^37^, ^38^ while others did not.^39^, ^40^, ^41^, ^42^, ^43^, ^44^, ^45^, ^46^, ^47^, ^48^, ^49^, ^50^, ^51^, ^52^, ^53^, ^54^, ^55^ At this time, no attempt has been made to systematically determine the impact of EID on RPE. Discrepancies between findings could potentially be related to methodological differences among studies, albeit this remains to be determined and confirmed with the aid of relevant analyses.

Efforts have yet to be deployed to determine, using a meta-analytic approach, the impact of EID on RPE. More specifically, there is a need to answer these questions: (1) does the change in RPE during exercise relate to EID?; (2) what is the magnitude of the effect of EID on RPE across different levels of EID; (3) is the magnitude of the effect of EID on RPE practically important?; (4) are there any identifiable factors among ambient temperature, humidity level, exercise intensity, exercise duration and aerobic capacity that may moderate the relationship between EID and RPE and; (5) to which extent “cardiovascular strain” mediates the relationship between EID and RPE? The goal of this study, therefore, is to use a meta-analytic approach to provide answers to the above-mentioned questions. Findings will be valuable to scientists, physical trainers, sports nutritionists, physicians, exercise physiologists, sports psychologists and any individuals engaged in regular exercise. Also, such findings will be valuable to elucidate whether the changes in body water are part of an integrated signal.

## 2 Methods

**Fig. 1:**
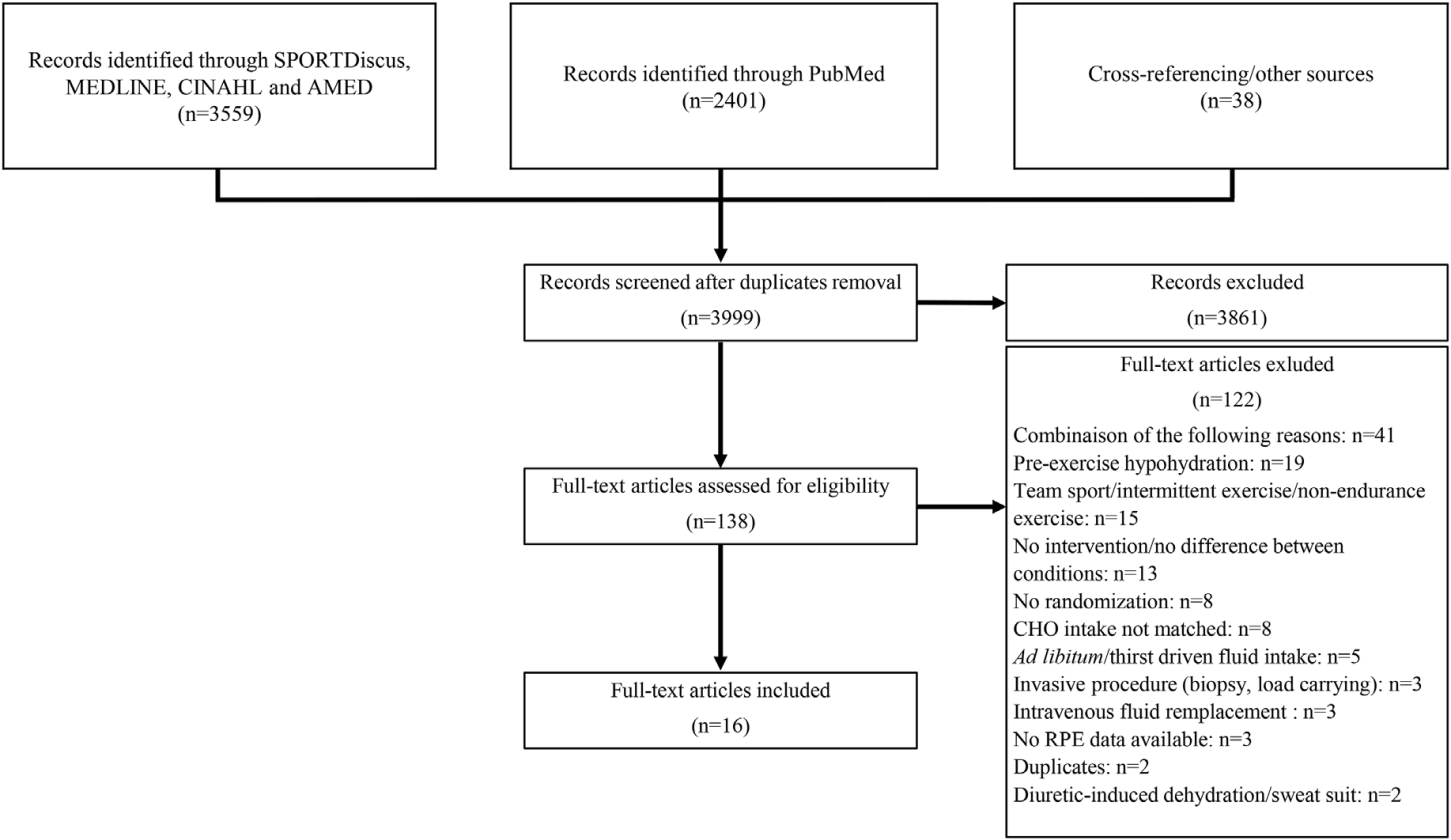
Flowchart showing the selection process used for the inclusion and exclusion of studies.

Fig. 1 reports the search strategy used for study selection. The literature search, limited to original peer-reviewed articles published in French or English, was performed with the PubMed, MEDLINE, SPORTDiscus, AMED and CINAHL databases, combining a “title field” and an “abstract field” research using the following keywords alone or in combination: cycling, dehydration, drink, effort, endurance, euhydration, exercise, exertion, fluid, hydration, hypohydration, perceived effort, perceived exertion, perception, performance, rate of perceived exertion, rating of perceived exertion, RPE and running. The exact search strategy can be found in supplementary material 1. A first selection based on the title was performed; afterward, the abstract and method sections of all potential articles were read. When hydration status was manipulated and RPE measured, the methodological section was carefully read to verify eligibility. Published abstracts, case studies, non-peer-review manuscripts and conference proceedings were not considered. Cross-referencing was performed on included studies and 6 narrative/systematic reviews.^24^^,^^56^, ^57^, ^58^, ^59^, ^60^ When needed, authors of included studies were contacted and asked to share experimental raw data. The last search of the literature was done on February 16, 2022. The review and the protocol were not registered. The meta-analysis was conducted using the Preferred Reporting Items for Systematic Reviews and Meta-analysis (PRISMA) guidelines.

### 2.1 Criteria for considering studies for inclusion/exclusion in the meta-analysis

Inclusion criteria were: (1) controlled study using a randomized crossover design in healthy adults (≥18 years old); (2) ≥30 min of continuous running or cycling endurance exercise (exercises <30 min are unlikely to induce substantial EID, or be limited by EID); (3) final EID in the experimental group >1% of pre-exercise body mass and ≥0.5% than the euhydrated (EUH) control condition; (4) dehydration progressively induced during, not before exercise^61^; (5) body mass change with EUH was within −1 to + 0.5% of the pre-exercise body mass^61^; (6) fluid replacement was given orally; (7) if carbohydrates (CHO) or caffeine were provided during exercise, the amount was identical between conditions^62^^,^^63^ and; (8) data required to compute changes in EID and RPE were included/available. Exclusion criteria were: (1) sports-specific and intermittent exercises; (2) use of diuretics or sweatsuits to accelerate EID; (3) provision of fluid according to thirst sensation; (4) uncontrolled ambient conditions or experimentation timing; (5) collection of muscle biopsies and; (6) carrying of loads during exercise.

### 2.2 Assessment of trial quality

No specific and validated tool to assess the quality of exercise-related studies has been developed. Moreover, assessing trial quality in meta-analyses using a scale can influence the interpretation of results.^64^ Hence, trial quality assessment was not performed in the present meta-analysis.

### 2.3 Data extraction

Using double data entry, data regarding (1) study characteristics; (2) participants characteristics; (3) exercise protocol characteristics; (4) EID levels and; (5) RPE were extracted and coded in spreadsheets. When not provided by authors, data only available in figures were extracted using WebPlotDigitizer.

### 2.4 Exercise duration and intensity and participants’ $\dot{V}$O_2max_

Exercise duration was computed as the average exercise time completed during both the EID and EUH conditions. Exercise intensity was taken as the average of the mean % $\dot{V}$O_2max_ at which both the EID and EUH conditions were performed. Mean exercise intensity was computed using a weighted average for those studies that used a combination of exercise intensities. When not measured by authors, exercise intensity was estimated and computed as explained by Goulet.^61^ Most studies^27^^,^^28^^,^^33^^,^^34^^,^^41^, ^42^, ^43^, ^44^^,^^46^^,^^47^^,^^65^, ^66^, ^67^, ^68^ reported participants’ $\dot{V}$O_2max_; Barwood, Goodall, Bateman^69^ and Dugas, Oosthuizen, Tucker, Noakes^40^ did not, and these values were calculated as in Goulet.^61^

### 2.5 Fluid intake, exercise-induced dehydration and dehydration rate measurement

Hydration rate (mL/min) was computed as the total amount of fluid intake divided by exercise duration, with the relative hydration rate (mL/min/kg) corrected for pre-exercise body mass (kg). The percent change in body mass from the pre-to post-exercise period was used as an index of the level of dehydration incurred during exercise. While this index is an imperfect representation of EID^70^ as it is impacted by both metabolic water production and gas exchange during exercise, measurement error is relatively low amounting to an overestimation of fluid loss of ∼100 mL/h during moderate intensity exercise.

When not provided, pre-exercise body mass was taken as that provided in the sample description, whereas % body mass loss was computed using the following equation:

(1) Pre-exercise body mass (kg) - post-exercise body mass (kg) / pre-exercise body mass (kg) x 100

Thus, any positive value represents a body mass loss while negative values indicate body mass gain.

Assuming a high repeatability of,^71^ and consistency in,^72^ sweating rate and thus body mass loss^73^ during exercise at a given intensity, dehydration rate (% body mass loss/min) was computed as follows:

(2) End of exercise body mass loss (%) / exercise duration (min)

**Fig. 2:**
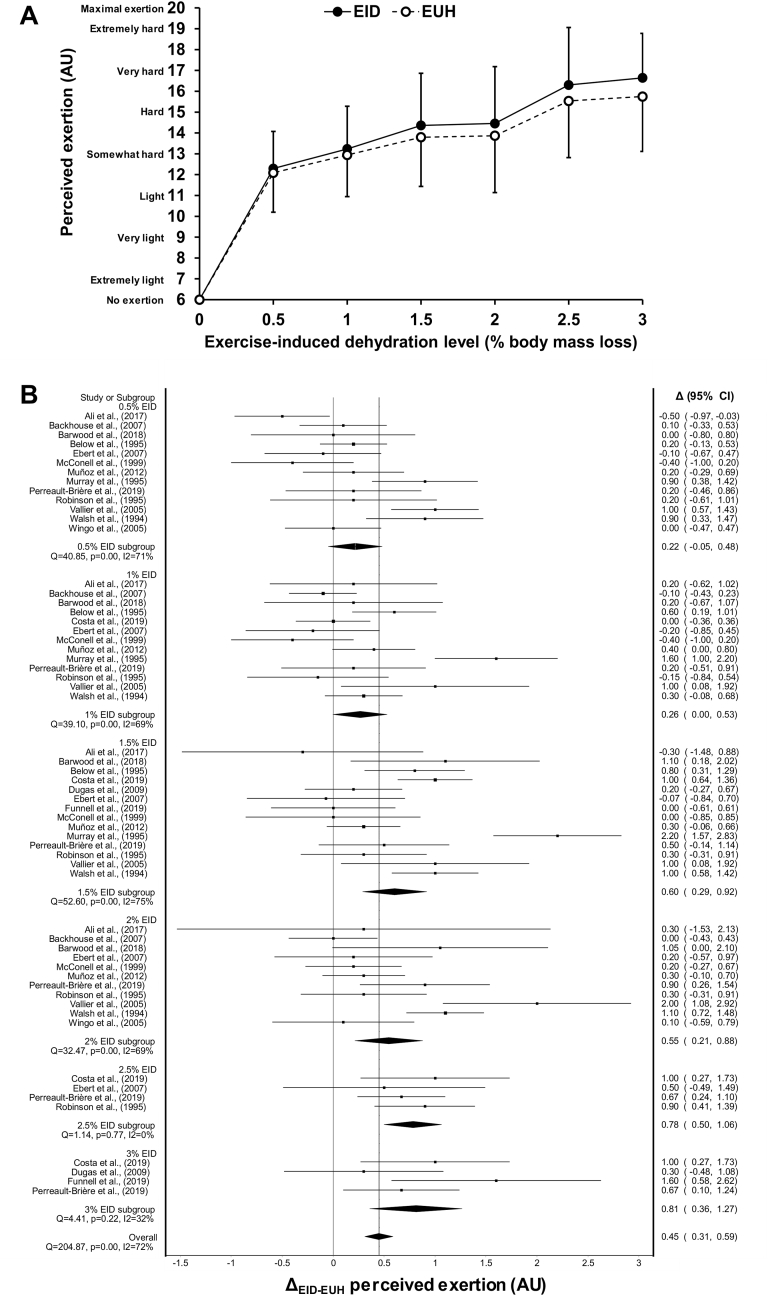
(a) Changes in perceived exertion (means ± SD) occurring during exercise between the euhydrated control condition and the exercise-induced dehydration (EID) experimental condition across levels of exercise-induced dehydration of 0.5, 1, 1.5, 2, 2.5 and 3% body mass. (b) Forest plot showing the mean differences in perceived exertion across different levels of exercise-induced dehydration. Filled diamond symbols represent the weighted mean change in perceived exertion between conditions. Size of squares is proportional to the weight of each study. AU: arbitrary units. CI: confidence interval.

Dehydration rate was used to calculate the % body mass loss associated with each measurement of RPE within a single study. For example, if in a study RPE was measured at 30, 60 and 90 min and the dehydration rate was 0.03%/min, therefore the corresponding % body mass losses were respectively taken as 0.9 (ex., 30 min × 0.03%/min), 1.8 and 2.7%. In some studies, this iteration process had to be stopped when body mass loss surpassed 1% in the EUH condition.^28^^,^^42^^,^^44^^,^^46^^,^^67^ This procedure enabled us to pinpoint the behavior of RPE across a wide range of % body mass loss changes, using research data available from all included studies. To provide a practical, easy to understand and clear visual characterization of the effect of body mass loss on RPE during exercise, Fig. 2a and b present the relationship between % body mass loss and RPE at fixed and anchored body mass loss levels, according to the classification presented in Table 1.

**Table 1:** Classification of percent body mass losses.

From	to	corresponds to
0.26	0.75%	0.5%
0.76	1.25%	1.0%
1.26	1.75%	1.5%
1.76	2.25%	2.0%
2.26	2.75%	2.5%
2.76	3.25%	3.0%

### 2.6 Measurement of perceived exertion during exercise

Perceived exertion data are presented according to the original 6–20 Borg scale. Dugas, Oosthuizen, Tucker, Noakes^40^ and Walsh, Noakes, Hawley, Dennis^33^ used the Borg-CR10 scale (range 0–10); for those studies, RPE was converted back to the 6–20 scale according to Arney, Glover, Fusco, Cortis, de Koning, van Erp, Jaime, Mikat, Porcari, Foster.^74^ For Barwood, Goodall, Bateman^69^ (hot and cold drink conditions), Dugas, Oosthuizen, Tucker, Noakes^40^ (0, 33 and 66% conditions) and Murray, Michael, McClellan^34^ (5- and 10-min conditions) merging of experiments were performed to eliminate data dependency.

### 2.7 Moderating variables

The following variables were *a priori* identified as potential moderators for the relationship between RPE and EID: ambient temperature, relative humidity, exercise duration and intensity and participants’ $\dot{V}$O_2max_. Ambient temperature and relative humidity are interdependent, as are exercise intensity and duration. To take this into account, absolute humidity and a composite score of exercise stress (product of exercise intensity (% $\;\dot{V}$O_2max_) and exercise duration (min)) were also considered as moderating variables.

### 2.8 Mediating variable

Heart rate was considered a potential mediating variable regarding the relationship between EID and RPE. Core temperature would have been another one to consider, but the paucity of data prevented us from evaluating its impact. For each study, mean exercise heart rate difference between the EUH and EID conditions was computed by averaging the sum of the heart rate difference computed at each measurement point.

### 2.9 Statistical analyses

#### 2.9.1 Software

Data were analyzed in their original form using Microsoft Office Excel 2020 (version 1902, Redmond, WA, USA), MetaXL (version 5.3, EpiGear), Comprehensive Meta-Analysis (version 2.2.064, Englewood, NJ, USA), STATA/MP (version 14, College Station, TX, USA), SPSS macros provided by Lipsey and Watson^61^^,^^75^ and IBM SPSS Statistics (version 21, Armonk, NY, USA) software.

#### 2.9.2 Weighted mean effect summaries

Each of the studies included in the meta-analysis took measurements of RPE during exercise at more than one EID level. Therefore, a meta-analysis of repeated measures was performed in an effort to limit the violation of the assumption of data independence in the data structure.^76^ First, an all-points forest plot was constructed to determine the mean effect of EID on RPE at body mass loss levels fixed and anchored at 0.5, 1, 1.5, 2, 2.5 and 3%. This method allows us to illustrate the rate of increase in RPE across this range of body mass losses. Moreover, it allows for more precision in establishing the relationship between RPE and body mass loss as this strategy increases the n for any dehydration point. All RPE data within a given EID level were independent of each other; however, each study contributed in providing RPE-related data to more than one EID level. Nevertheless, the assumption of independence was protected for each of the EID levels. Post-hoc analyses were done using the False Discovery Rate procedure, with the number of *a priori* defined comparisons taken as 6, mirroring each of the EID levels compared. Second, to establish the mean effect of EID on RPE for each increase in 1% body mass loss, a forest plot was constructed from the slope estimates of the relationships between EID and RPE for each of the included studies, using non-weighted linear regression analyses with the intercepts forced through the origin.^76^ For this forest plot, n was taken as the number of EID levels included in the regression analysis. Initially, the analyses were performed separately by subgroups. However, data from studies using time-trial type exercise protocols were combined with those using fixed-intensity exercise protocols given the low number of studies using time-trial type exercise and because variations in RPE within the different EID levels were similar and, in all instances, <1 point. Nonetheless, on few occasions, analyses excluding time-trial type exercises will be presented when deemed interesting. Weighted mean effect summaries were determined using method of moment random-effects model. A more intuitive approach was used to verify whether it would change the outcomes in comparison to our approach. For that, we averaged, within a given hydration condition (EUH and EID), all RPE measurements across time, and then observed the difference in RPE between conditions.

#### 2.9.3 Practical significance of the weighted mean effect summaries and slope estimate

The qualitative interpretation of the practical significance of the effect of EID on RPE was performed as in Goulet & Hoffman.^59^ Previous studies observed reliability of the RPE scale to be <1 point.^77^^,^^78^ Because the minimal increment of the scale is 1 point, this threshold was taken and accepted as the smallest worthwhile practical difference in RPE.

#### 2.9.4 Heterogeneity, publication bias and sensitivity analysis

Cochran's *Q* and *I*^2^ statistic were both used to assess between-study heterogeneity and the degree of inconsistency among results of included studies. Cochran's Q test was considered significant at *p* ≤ 0.1. ^79^ The following classification was used to interpret the I^2^ statistic: low (<40%), moderate (40–59%), substantial (>60%).^80^ Publication bias was performed using visual assessment of funnel plots with Trim and Fill adjustments. A sensitivity analysis was performed on each of the forest plots by removing each study once from the models to determine whether this would change the magnitude of the outcome summaries.

#### 2.9.5 Meta-regression analyses

The potential mediating effect of heart rate and moderating effect of the *a priori* defined confounders were determined by regressing the slope estimates upon the mean heart rate difference between conditions or each of the confounders, respectively. Confounder and mediator variables included at least 10 data points from 10 different studies. A multiple meta-regression combining all moderators (with the exception of absolute humidity and composite score of exercise stress) was performed to understand the strength of our proposed model. Meta-regression analyses were performed using method of moment random-effects model, with 95% robust (Huber-Eicher-White-sandwich) standard errors.

#### 2.9.6 Statistical significance

Otherwise stated, in all instances, results were considered significant at *p* < 0.05 or when the 95% confidence interval did not include 0.

#### 2.9.7 Variance computations

When raw data were obtained from the authors, variances were directly calculated from the Δ standard errors or standard deviations of the absolute changes in RPE between conditions. Otherwise, individual variances for changes in RPE were estimated as in Goulet, Hoffman^59^ using an imputed weighted correlation coefficient of 0.81 deriving from 40 correlation coefficients obtained from 5 different studies whose authors provided raw data.

## 3 Results

### 3.1 Search results and characteristics of the included studies

After removing duplicates, 3999 titles were checked (Fig. 1). In the remaining 138 articles assessed for eligibility, 16 were included in the meta-analysis (Table 2). The studies were published between 1994 and 2019 in 10 different peer-reviewed journals. Four studies were conducted in the USA,^27^^,^^34^^,^^42^^,^^68^ 3 in the UK,^28^^,^^46^^,^^69^ Australia,^41^^,^^44^^,^^66^ and South-Africa^33^^,^^40^^,^^47^ and 1 in Canada,^43^ France^67^ and New-Zealand.^65^

**Table 2:** Summary of characteristics of included studies.

References	Participants: n (women), age (years), $\dot{V}$O_2max_ (mL/kg/min)	Protocol: total duration (min), exercise mode, temperature (°C), relative humidity (%), wind speed (km/h), fluid temperature (°C)	Fluid intake (mL/kg/min)	Same time of the day, familiarisation, same diet before	Body mass loss (%)^$^, dehydration rate (% body mass loss/min)^$^	RPE measurement and conclusion
**Studies that evaluated perceived exertion during fixed intensity exercise**						
Backhouse et al. (2007) ^46^	15 (0) endurance-trained, 21, 65	90, running at 70% $\dot{V}$O_2max_, 20, 47, 0, 10 Only 60 min included in the analysis because body mass loss >1% at 80 min in EUH^#^.	EUH: 0.14 EID: 0.0	Yes, no, yes	At the end of 90 min: EUH: 1.4, 0.016 EID: 2.7, 0.030 At 60 min^#^: EUH: 0.9 EID: 1.8	Borg 6-20 scale, reported every 20 min. No differences.

Barwood et al. (2018) ^69^	10 (0) non-heat acclimatized trained cyclists, 25, 60∗	60+5, cycling at 55% P_max_ (59% $\dot{V}$O_2max_) followed by 80% P_max_ (90% $\dot{V}$O_2max_) time to exhaustion, 34, 34, 10.1, 27	EUH: 0.20 EID: 0.0	Yes, no, yes	At the end of the performance: EUH: 0.9, 0.014 EID: 2.1, 0.033	Borg 6-20 scale, reported every 15 min. No differences. Raw data provided by authors.

Below et al. (1995) ^27^	8 (0) endurance trained, 23, 63	50+11, cycling at 80% of $\dot{V}$O_2max_ followed by a performance test at 79% $\dot{V}$O_2max_, 31, 54, 12.6, 38 Same CHO intake	EUH: 0.31 EID: 0.05	Yes, yes, yes	At the end of the performance: EUH: 0.5, 0.008 EID: 1.9, 0.031	Borg 6-20 scale, reported every 10 min. RPE only significantly higher in EID at 40 and 50 min.

Costa et al. (2019) ^66^	11 (0) competitive endurance runners, 34, 59	120, running at 70% $\dot{V}$O_2max_, 25, 46, 10.6, 24.7	EUH: 0.18 EID: 0.0	Yes, no, yes	EUH: 0.6, 0.005 EID: 3.1, 0.026	Borg 6-20 scale, reported every 30 min. RPE only significantly higher in EID at 120 min.

Ebert et al. (2007) ^44^	8 (0) well-trained cyclists, 28, 66	120+17, cycling at 53% P_max_ (55% $\dot{V}$O_2max_) followed by cycling hill-climb time-to-exhaustion trial at 88% P_max_ (85% $\dot{V}$O_2max_), 29, 37, 15, -Same CHO intake Only 120 min included in the analysis because body mass loss >1% at the end of the performance in EUH^#^.	EUH: 0.28 EID: 0.05	Yes, yes, yes	At the end of the 120 min: EUH: -0.3, -0.0025 EID: 2.5, 0.021	Borg 6-20 scale, reported every 15 min. No differences. Raw data provided by authors.

Funnell et al. (2019) ^28^ Only unblinded group	7 (0) trained, non-heat acclimated cyclists/triathletes, 26, 64	120+15, cycling at 50% P_max_ (51% $\dot{V}$O_2max_) followed by a time-trial (77% $\dot{V}$O_2max_), 31, 48, 21.2, 37 Only 120 min included in the analysis because body mass loss >1% at the end of the performance in EUH^#^.	EUH: 0.23 EID: 0.02	Yes, yes, yes	At the end of the 120 min: EUH: 0.5, 0.004 EID: 3.0, 0.025	Borg 6-20 scale, reported at 60 and 120 min. RPE only significantly higher in EID at 120 min. Raw data provided by authors.

McConell et al. (1999) ^41^	8 (0) well-trained cyclists and triathletes, 26, 64	45+15, cycling at 80% of $\dot{V}$O_2max_ followed by an "all-out" performance at 77% $\dot{V}$O_2max_, 21, 41, wind but speed not reported, room temperature	EUH: 0.31 EID: 0.0	Yes, yes, yes	At the end of the performance: EUH: 0.0, 0.0 EID: 1.9, 0.032	Borg 6-20 scale, reported at 10, 30, 45 and 60 min. No differences.

Muñoz et al. (2012) ^42^	10 (0) healthy runners, 25, 60	90+23, running at 30% $\dot{V}$O_2max_ followed by a 5 km time-trial (72% $\dot{V}$O_2max_), 33, 30, 0, 7 Only 90 min included in the analysis because body mass loss >1% at the end of the performance in EUH^#^.	EUH: 0.12 EID: 0.0	Yes, yes, yes	At the end of 90 min: EUH: 0.9, 0.010 EID: 1.8, 0.020	Borg 6-20 scale, reported every 5 min. No differences. Raw data provided by authors.

Murray et al. (1995) ^34^	15 (0) trained, 29, 50	60, cycling at 50% $\dot{V}$O_2max_, 32, 70, 0, 5	EUH: 0.35 EID: 0.0	Yes, no, -	EUH: 0.1, 0.002 EID: 1.7, 0.028	Borg 6-20 scale, reported every 5 min. RPE significantly higher in EID than EUH (5 min condition) from 30 to 60 min. No difference between EID and EUH (10 min condition).

Vallier et al. (2005) ^67^	8 (0) competitive trained cyclists or triathletes, 31, 63	180, cycling at 60% $\dot{V}$O_2max_, 20.5, 50, 9, 18.5 Only 80 min included in the analysis because body mass loss >1% at 80 min in EUH^#^.	EUH: 0.17 EID: 0.0	Yes, no, yes	At the end of the 180 min: EUH: 2.2, 0.012 EID: 4.1, 0.023 At 80 min^#^: EUH: 1.0 EID: 1.8	Borg 6-20 scale, reported every 20 min. No differences. However, the increase in RPE appears earlier in EID (100 min) compared to EUH (120 min).

Walsh et al. (1994) ^33^	6 (0) endurance trained competitive cyclists or triathletes, 26, 61	60+8, cycling at 70% $\dot{V}$O_2max_ followed by a time to exhaustion at 90% $\dot{V}$O_2max_, 30, 60, 3, 5	EUH: 0.23 EID: 0.0	Yes, no, yes	At the end of the 60 min: EUH: 0.2, 0.0033 EID: 1.8, 0.03	Borg CR10 scale, reported every 10 min. RPE higher in EID at 60 min only.

Wingo et al. (2005) ^68^	9 (0) trained cyclists, 25, 55	45+7, cycling at 64% $\dot{V}$O_2max_ followed by maximal test at 78% $\dot{V}$O_2max_, 35, 40, 0, 35	EUH: 0.47 EID: 0.0	Yes, yes, -	At the end of the performance: EUH: 0.3, 0.006 EID: 2.5, 0.049	Borg 6-20 scale, reported at 15 and 45 min. No differences. Raw data provided by authors.
**Studies that evaluated perceived exertion during self-paced intensity exercise**						
Ali et al. (2017) ^65^	9 (0) moderately trained cyclists, 33, 55	68.5, cycling time-trial at 78% $\dot{V}$O_2max_, 19, 48, 0, 6	EUH: 0.11 EID: 0.0	Yes, yes, yes	EUH: 0.6, 0.009 EID: 1.9, 0.028	Borg 6-20 scale, reported every 25% of exercise completed (every 17.1 min). No differences. Raw data provided by authors.

Dugas et al. (2009) ^40^	6 (0) highly trained cyclists, 23, 77∗	127, 80 km cycling time-trial at 47% P_max_ (50% $\dot{V}$O_2max_), 33, 50, 37.5, -Same CHO intake	EUH: 0.32 EID: 0.10	Yes, yes, yes	EUH: 0.5, 0.004 EID: 3.0, 0.024	Borg CR10 scale, reported at 40 and 80 km (at 63 and 127 min). No differences.

Perreault-Brière et al. (2019) ^43^	9 (2) heat- or partially heat-acclimatized, healthy, endurance-trained competitive cyclists and triathletes, 30, 59 Women tested during the follicular phase.	60, cycling time-trial at 80% $\dot{V}$O_2max_, 30, 49, 27.5, 5	EUH: 0.37 EID: 0.0	Yes, yes, yes	EUH: 0.6, 0.010 EID: 2.9, 0.048	Borg 6-20 scale, reported every 5 min. No differences. Raw data provided by authors.

Robinson et al. (1995) ^47^	8 (0) endurance-trained cyclists, 25, 66	60, cycling time-trial at 79% $\dot{V}$O_2max_, 20, 60, 10.8, 5	EUH: 0.32 EID: 0.0	Yes, yes, yes	EUH: 0.9, 0.016 EID: 2.3, 0.038	Borg 6-20 scale, reported every 10 min. No differences.

References are listed in alphabetical order for both sections. EID: exercise-induced dehydration (experimental condition), EUH: euhydration (control condition), RPE: perceived exertion, -: missing data, ∗: estimated $\dot{V}$O_2max_ using Hawley & Noakes (1992) equations. Value of 0 was attributed when wind speed was not provided. #: indicates the % of body mass loss taken for analysis for those studies in which body mass loss surpassed 1% in the EUH condition. $: any positive value represents a body mass loss while negative values indicate body mass gain.

### 3.2 Participant's characteristics

A total of 147 endurance-trained individuals are represented among the 16 included studies, with women representing only 1% of the total sample. Mean sample size was 9 ± 3 individuals per study (range 6–15). None of the included studies reported information about ethnicity. The mean age, height, body mass, body mass index, $\dot{V}$O_2max_ and peak power output of the participants were respectively 27 ± 4 years, 179 ± 2 cm, 73 ± 3 kg, 23 ± 1 kg/m^2^, 62 ± 6 mL/kg/min and 389 ± 39 W.

### 3.3 Characteristics of the exercise protocols

Among the selected studies, 81% (n = 13) used cycling as the mode of exercise while the remaining used running (n = 3). The mean ambient temperature and relative humidity were respectively 28 ± 6 °C and 48 ± 10%, with a mean wind speed of 10 ± 11 km/h. The mean exercise duration and intensity were respectively 79 ± 27 min (range 51–127 min) and 65 ± 13% of $\dot{V}$O_2max_.

### 3.4 Fluid consumption and exercise-induced dehydration levels

Mean rates of fluid consumption in the EUH and EID conditions were respectively 18.9 ± 7.5 and 1.0 ± 2.0 mL/min, representing 0.26 ± 0.1 and 0.01 ± 0.03 mL/kg/min. The average fluid temperature was 17 ± 13 °C. The mean end-of-exercise body mass loss was 0.5 ± 0.4% (range 1 to −0.3%) when EUH was attempted to be maintained, compared to 2.3 ± 0.5% (range 1.7–3.1%) with EID, for a mean difference of 1.7 ± 0.7% (range 0.9–2.8%) between conditions. Mean dehydration rates of 0.007 ± 0.005 and 0.03 ± 0.009%/min were observed during the EUH and EID conditions, respectively.

### 3.5 Weighted mean effect summaries

Fig. 2a depicts the changes in RPE that occurred during exercise between the EUH and EID conditions across levels of body mass losses of 0.5, 1, 1.5, 2, 2.5 and 3%, and for absolute values of RPE which fluctuated from ∼12 (light) to 16.5 (hard/very hard) points. Fig. 2b pinpoints the weighted mean difference in RPE between the EUH and EID conditions across each of these levels of body mass losses. Results of the forest plot illustrate that, compared with EUH, EID slowly increased RPE during exercise from 0.22 points (95% CI: -0.05–0.48) when body mass loss was trivial (0.5%) to 0.60 points (95% CI: 0.29–0.92) when body mass loss was light (1.5%), up to 0.81 points (95% CI: 0.36–1.27) when body mass loss was moderate (3%). Only at 0.5 and 1% body mass losses were the differences in RPE between the EUH and EID conditions not significant. In none of the 6 weighted mean summary effects models did the removal of each study one at a time significantly and practically impacted the outcome that body mass loss has upon RPE. For each of the EID subgroups, the practical impact of body mass loss on RPE was likely or almost certainly trivial. Distribution of point estimates around each of the 6 weighted mean effect summaries was appropriate, which indicates no publication bias. Cluster analysis indicates that heterogeneity was substantial with an *I*^2^ of 72% and a Cochran's *Q* of 204.9, *p* < 0.01. At the subgroup level, substantial inconsistencies were also observed at the 0.5, 1, 1.5 and 2, but not 2.5 and 3% body mass loss levels where heterogeneity was low. When the analyses were performed without the studies that used time-trials, results were similar, with the exception that the differences in RPE between the EUH and EID condition reached 1.2 points (95% CI: 0.61–1.80) when body mass loss was moderate (3%).

Cluster analysis indicates that EID, on average, increases RPE by 0.45 point (95% CI: 0.31–0.59, Fig. 2b). Using the more intuitive approach, we observed an overall effect of 0.38 points (95% CI: 0.22–0.53, *Q* = 125.3, *p* < 0.01, *I*^2^ = 88%). When studies that used time-trials were removed, the results were, again, extremely similar: 0.50 points (95% CI: 0.33–0.67, *Q* = 174.8, *p* < 0.01, *I*^2^ = 77%) *vs.* 0.44 points (95% CI: 0.24–0.64, *Q* = 114.2, *p* < 0.01, *I*^2^ = 90%) with the more intuitive approach.

**Fig. 3:**
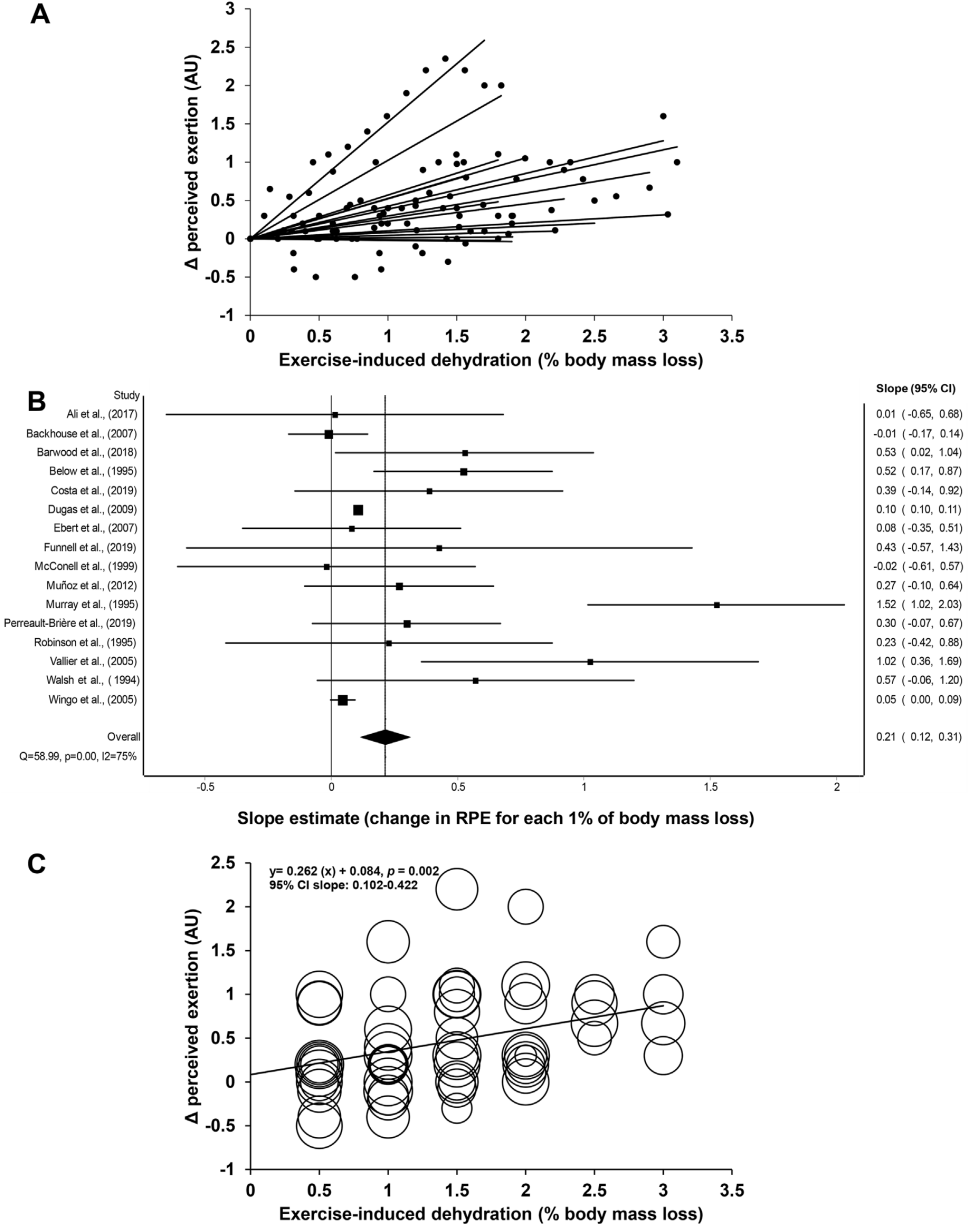
(a) Slope estimates for the regression of perceived exertion on the % body mass loss for each of the included studies; (b) Forest plot combining all slope estimates to derive a weighted mean summary effect; (c) Relationship between perceived exertion and % body mass loss while including all study-specific data points. AU: arbitrary units. CI: confidence interval. RPE: rating of perceived exertion.

Fig. 3a illustrates the slope estimates for the regression of RPE on the % body mass loss for each of the included studies. Fig. 3b shows a forest plot combining the 16 slope estimates to derive a weighted mean summary effect. Results show that for each 1% body mass loss, RPE increased on average by 0.21 points (95% CI: 0.12–0.31), thereby theoretically suggesting that it is not before reaching a body mass loss of ∼5% that EID may potentially affect RPE in a meaningful way. A sensitivity analysis revealed that the removal of each study one at a time from the model did not significantly nor practically alter the outcome of the weighted mean effect summary, with variations in the slope estimate ranging from 0.13 (95% CI: 0.06–0.20) to 0.35 points (95% CI: 0.17–0.53). Inconsistency among research observations was substantial with an *I*^2^ of 75% and a Cochran's *Q* of 58.99, *p* < 0.01. Point estimates were not equally distributed on each side of the weighted mean summary effect, thereby suggesting publication bias. A trim and fill analysis adjusting for missing studies at the left side of the mean changed the weighted mean effect summary to 0.10 points (95% CI: 0.00–0.22). When the analyses were performed without the studies that used time-trials, results showed that for each 1% body mass loss, RPE increased on average by 0.38 points (95% CI: 0.17–0.59, *Q* = 56.2, *p* < 0.01, *I*^2^ = 80%). Fig. 3c depicts the relationship between RPE and % body mass loss while including all 59 study-specific data points, which violates the assumption of independence among data. Nevertheless, the weighted regression analysis provides a slope estimate (0.26 points, 95% CI: 0.10–0.42) which is congruent to the one built from combining all 16 slope estimates.

### 3.6 Meta-regression analyses

**Fig. 4:**
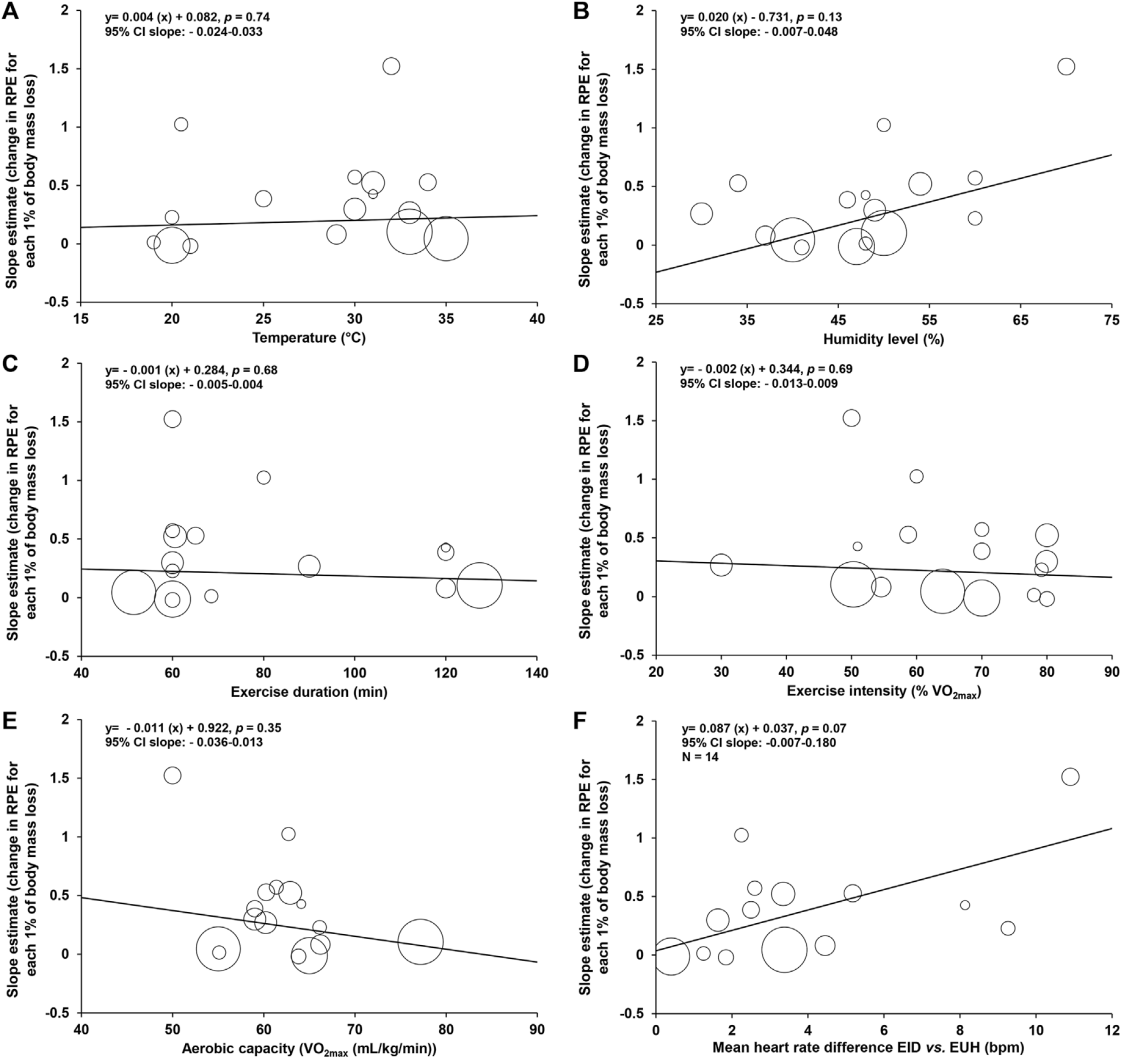
Relationships between the changes in slope estimates and (a) temperature, (b) humidity level, (c) exercise duration, (d) exercise intensity, (e) aerobic capacity ($\dot{V}$O_2max_) and mean (f) heart rate difference across the different studies included in the meta-analysis. Otherwise stated n = 16. CI: confidence interval. EID: exercise-induced dehydration (experimental condition). EUH: euhydration (control condition). RPE: rating of perceived exertion.

Fig. 4 shows the relationships between the changes in slope estimates and (a) temperature, (b) humidity level, (c) exercise duration, (d) exercise intensity, (e) aerobic capacity and (f) mean heart rate difference across the different studies. Individually, none of these variables significantly correlated to the extent of changes in RPE for each 1% in body mass loss. When all these variables were combined in a multiple meta-regression model (except heart rate), the goodness of fit reached 66%. The same picture was observed using the model derived from of all 59 study-specific data points. No significant relationships were also observed between the changes in slopes estimates and absolute humidity (*p* = 0.26) or the composite score of exercise stress (*p* = 0.65).

## 4 Discussion

Despite that exercisers have access to a large and diversified arsenal of tools to monitor exercise intensity, the ability to maintain a certain speed or power output is, ultimately, tributary to RPE.^8^, ^9^, ^10^ On the other hand, enjoyment of exercise is an important component of participation and adherence, and the more the exercise is perceived to be strenuous, the less likely the exercise behaviour is to be maintained.^12^ In a sense, therefore, RPE can be considered as the ‘’mastermind’’ of exercise performance or adherence. Consequently, factors susceptible to negatively impact the sense of effort during exercise should be given particular attention. Albeit the current results do show that EID increases RPE (response to question #1), the effect was shown to be below our identified threshold of 1 point, at least for the included studies where the greatest level of EID reached was 3% (response to questions #2 and #3), and not moderated by key confounders or associated with changes in heart rate (response to question #4 and #5). Therefore, our results highlight for the first time that EID <3% of body mass has a spurious effect on RPE, contrarily to what is believed.

It has been suggested that RPE may act as a mediator of the effect of EID on endurance performance.^14^^,^^24^ This is legitimate from a physiological perspective, as the documented impact of EID on thermoregulatory, cardiovascular and metabolic functions^14^^,^^24^ should result in a higher perceived strain and, hence, RPE. However, our results including observations from cycling and running exercises conducted at clamped and self-paced intensities and under different environmental conditions show that although the change in RPE statistically relates to EID, the magnitude of the effect is unlikely to be practically meaningful until a body mass of at least 3%. Of course, the design of the present meta-analysis precludes from inferencing about the repercussion of the EID-induced increase in RPE on endurance performance. Nevertheless, we are aware of no studies which have been able to establish a decisive relationship between RPE and endurance performance. That being said, if scientists agree upon the simplistic and imperfect model indirectly linking the EID-induced increase in RPE with the decline of endurance performance, the present findings clearly dispute this assertion, at least for EID <3% of body mass.

It is proposed that EID may reduce exercise participation/adherence in recreationally active individuals because of its impact upon RPE.^25^^,^^81^ Based on urinary indices, studies have suggested that 40–50% of recreationally active individuals begin exercise in a light hypohydration state^25^^,^^82^ and lose ∼0.6% of their body mass while drinking fluid *ad libitum* during freely chosen exercise sessions.^25^ Furthermore, untrained individuals have lower sweat rate^19^^,^^83^ and generally an easy access to water during exercise. Regarding women, they generally have a lower sweat rate than men and drink more (sometimes more than their sweat losses) during exercise.^84^^,^^85^ Therefore, it is unlikely that those individuals will reach EID levels ≥3% of body mass during typical physical activities and, hence, that the EID-associated increase in RPE should not be a cause for concern. Moreover, studies have reported worsened affective response when participants begin exercise in a low hypohydration state^25^ or when dehydration occurs progressively during exercise.^46^ And it seems that the acute affective response to exercise more than the acute change in RPE relates to long-term adherence to exercise.^26^^,^^86^

Although it is agreed upon that the brain is the organ responsible for the regulation of RPE, whether it is centrally derived and largely independent of peripheral afferent signals^4^^,^^87^, ^88^, ^89^ or results from the integration and interpretation of afferent feedback from the peripheral machinery^5^^,^^90^, ^91^, ^92^ is debated (see^6^^,^^93^, ^94^, ^95^ for further details). If the first theory is favoured, then it follows that EID would likely not alter the ability of the brain to produce motor forward commands, termed efference copies or corollary discharges.^96^ It has been shown that acute EID and the associated hyperosmolality does not alter brain volume,^97^ potentially highlighting the fact that the cerebral cortex operates close to optimally under dehydrating conditions. On the other hand, interpreting the current results within the context of the second theory would imply that EID provokes minimal homeostasis alterations, at least up to a body mass loss of 3%. This claim is reasonable given that the EID-induced increase in heart rate (∼4 beats/min, n = 14) and core temperature (∼0.2 °C, n = 9) (data directly extracted from the included studies of the present meta-analysis) was marginal compared with EUH.

There was substantial heterogeneity among research findings, as illustrated in Fig. 3b. This is unremarkable given that studies used a variety of protocols within which factors known to influence RPE were present. Meta-regressions were conducted to examine the moderating effect of *a priori* selected confounders. They showed that, in isolation, neither humidity, exercise duration, exercise intensity, aerobic capacity nor ambient temperature significantly correlated to the extent of changes in RPE for each 1% of body mass loss. However, while aggregated into one model, those 5 variables explained 66% of the variance observed among the changes in RPE for each 1% of body mass loss. This figure is impressive and depicts the importance of the identified confounders in the overall moderation of the relationship between EID and RPE. Hence, it cannot be excluded that the significance of several influential variables within each of the included studies masked the ability to clearly identify the independent moderating effect of some or all of the confounders.

There was a trend for the change in heart rate to be associated with the change in RPE for each 1% of body mass loss. Such an observation was to be expected because EID is known to exacerbate heart rate^60^ and the latter has been reported to be closely related to RPE.^90^ Indeed, the 6–20 Borg scale was initially developed in healthy individuals to correlate approximately with exercise heart rate. Roughly, our model indicates that for each increase in heart rate of 1 beat/min there should be an increase in RPE of 0.1 points for each 1% of body mass loss. Providing that the mean change in heart rate during exercise was <10 beats/min between EUH and EID, our observation of a lack of a meaningful effect of EID on RPE makes sense. Nevertheless, it is important to bear in mind that the relationship between heart rate and RPE is only correlational in nature, not causal.^90^ Indeed, research has shown that it is possible to dissociate the change in heart rate from the change in RPE (e.g., using pharmacological agents).^98^^,^^99^ Therefore, our observation of a close relationship between the changes in heart rate and RPE for a given body mass loss should not be taken as a possibility that heart rate could act as a mediator of the relationship between EID and RPE.

Results of this meta-analysis must be interpreted with the following considerations or limitations in mind. The literature search was limited to English and French citations; studies published in different languages may have been missed. Similarly, in the literature of concern, RPE is almost exclusively studied as a secondary outcome. This complicates study identification which may have led us to miss key studies. Validity of RPE measurement depends upon the proper instructions provided to participants^93^; no studies reported on whether they dispensed such instructions. Little information is available regarding what represents a meaningful change in RPE; therefore, having used a different threshold may have modified our conclusions. In the present meta-analysis, women represented only 1% of the total sample. Recent evidence suggests that women may be more sensitive to the negative thermoregulatory effects of dehydration. Therefore, it could not be excluded that the relationship between EID and RPE may be different in women.^17^^,^^18^^,^^100^ While they would have provided insight into the possible mechanisms linking EID to RPE, data such as thirst sensation, changes in plasma osmolality and volume were not considered as they were reported by too few included studies. Having studies with higher level of EID (i.e. > 3% of body mass) would have provided more information and possibly modified our conclusions. Finally, the present results apply to adults, primarily males, up to an EID level of 3% body mass and for exercise up to ∼2 h in duration in thermoneutral to warm environments.

In conclusion, while from a statistical point of view, EID >1% of body mass increases RPE, the present results suggest that its effect is unlikely to be practically meaningful under running or cycling exercise conditions until a body mass loss of at least 3% is reached.

## 5 Perspectives

Perceived exertion can be considered as the ‘’mastermind’’ of exercise performance or adherence. Exercise-induced dehydration is generally thought to increase RPE. The greater RPE associated with EID may contribute to reducing (1) exercise performance in athletes and (2) exercise participation/adherence in recreationally active individuals. Findings of the present meta-analysis suggest that from a statistical point of view, EID >1% of body mass increases RPE. However, the effect of EID on RPE is unlikely to be practically meaningful under running or cycling exercise conditions, even at 3% of body mass loss. Thus, our results suggest that concerns about the impact of EID upon RPE and, thus, by extension, the effect of the latter on endurance performance or exercise participation/adherence seem not warranted, at least not until a body mass loss of 3%.

## References

[1] Riebe D., Ehrman J.K., Liguori G., Magal M., Medicine ACoS (2018).

[2] Borg G. (1970). Perceived exertion as an indicator of somatic stress. Scand J Rehabil Med.

[3] Borg G.A. (1982). Psychophysical bases of perceived exertion. Med Sci Sports Exerc.

[4] Marcora S. (2009). Perception of effort during exercise is independent of afferent feedback from skeletal muscles, heart, and lungs. J Appl Physiol.

[5] Robertson R.J., Noble B.J. (1997). Perception of physical exertion: methods, mediators, and applications. Exerc Sport Sci Rev.

[6] Abbiss C.R., Peiffer J.J., Meeusen R., Skorski S. (2015). Role of ratings of perceived exertion during self-paced exercise: what are we actually measuring?. Sports Med.

[7] Thiel C., Pfeifer K., Sudeck G. (2018). Pacing and perceived exertion in endurance performance in exercise therapy and health sports. German J Exercise Sport Res.

[8] Noakes T.D. (2008). Rating of perceived exertion as a predictor of the duration of exercise that remains until exhaustion. Br J Sports Med.

[9] Crewe H., Tucker R., Noakes T.D. (2008). The rate of increase in rating of perceived exertion predicts the duration of exercise to fatigue at a fixed power output in different environmental conditions. Eur J Appl Physiol.

[10] Eston R., Faulkner J., St Clair Gibson A., Noakes T., Parfitt G. (2007). The effect of antecedent fatiguing activity on the relationship between perceived exertion and physiological activity during a constant load exercise task. Psychophysiology.

[11] Ekkekakis P., Lind E. (2006). Exercise does not feel the same when you are overweight: the impact of self-selected and imposed intensity on affect and exertion. Int J Obes.

[12] Emad M., Neumann D.L., Abel L. (2017).

[13] Barnes K.A., Anderson M.L., Stofan J.R. (2019). Normative data for sweating rate, sweat sodium concentration, and sweat sodium loss in athletes: an update and analysis by sport. J Sports Sci.

[14] Cheuvront S.N., Kenefick R.W., Montain S.J., Sawka M.N. (2010). Mechanisms of aerobic performance impairment with heat stress and dehydration. J Appl Physiol.

[15] Montain S.J., Coyle E.F. (1992). Influence of graded dehydration on hyperthermia and cardiovascular drift during exercise. J Appl Physiol.

[16] Gonzalez-Alonso J., Mora-Rodriguez R., Below P., Coyle E. (1995). Dehydration reduces cardiac output and increases systemic and cutaneous vascular resistance during exercise. J Appl Physiol.

[17] Logan-Sprenger H.M., Heigenhauser G.J.F., Jones G.L., Spriet L.L. (2013). Increase in skeletal-muscle glycogenolysis and perceived exertion with progressive dehydration during cycling in hydrated men. Int J Sport Nutr Exerc Metabol.

[18] Logan-Sprenger H.M., Heigenhauser G.J.F., Killian K.J., Spriet L.L. (2012). Effects of dehydration during cycling on skeletal muscle metabolism in females. Med Sci Sports Exerc.

[19] Merry T., Ainslie P., Cotter J. (2010). Effects of aerobic fitness on hypohydration-induced physiological strain and exercise impairment. Acta Physiol.

[20] Goodman S.P., Marino F.E. (2021). Thirst perception exacerbates objective mental fatigue. Neuropsychologia.

[21] Armstrong L.E., Ganio M.S., Casa D.J. (2012). Mild dehydration affects mood in healthy young women. J Nutr.

[22] Ganio M.S., Armstrong L.E., Casa D.J. (2011). Mild dehydration impairs cognitive performance and mood of men. Br J Nutr.

[23] Goodman S.P., Moreland A.T., Marino F.E. (2019). The effect of active hypohydration on cognitive function: a systematic review and meta-analysis. Physiol Behav.

[24] James L.J., Funnell M.P., James R.M., Mears S.A. (2019). Does hypohydration really impair endurance performance? Methodological considerations for interpreting hydration research. Sports Med.

[25] Peacock O.J., Stokes K., Thompson D. (2011). Initial hydration status, fluid balance, and psychological affect during recreational exercise in adults. J Sports Sci.

[26] Williams D.M., Dunsiger S., Ciccolo J.T., Lewis B.A., Albrecht A.E., Marcus B.H. (2008). Acute affective response to a moderate-intensity exercise stimulus predicts physical activity participation 6 and 12 months later. Psychol Sport Exerc.

[27] Below P.R., Mora-Rodriguez R., Gonzalez-Alonso J., Coyle E.F. (1995). Fluid and carbohydrate ingestion independently improve performance during 1 h of intense exercise./L ' ingestion d ' hydrate de carbone et de boisson augmente independamment la performance pendant 1h dexercice physique intense. Med Sci Sports Exerc.

[28] Funnell M.P., Mears S.A., Bergin-Taylor K., James L.J. (2019). Blinded and unblinded hypohydration similarly impair cycling time trial performance in the heat in trained cyclists. J Appl Physiol.

[29] Ishijima T., Hashimoto H., Satou K., Muraoka I., Suzuki K., Higuchi M. (2009). The different effects of fluid with and without carbohydrate ingestion on subjective responses of untrained men during prolonged exercise in a hot environment. J Nutr Sci Vitaminol.

[30] Fallowfield J.L., Williams C., Booth J., Choo B.H., Growns S. (1996). Effect of water ingestion on endurance capacity during prolonged running. J Sports Sci.

[31] Mudambo K.S.M.T., Leese G.P., Rennie M.J. (1997). Dehydration in soldiers during walking/running exercise in the heat and effects of fluid ingestion during and after exercise. Eur J Appl Physiol Occup Physiol.

[32] Barr S.I., Costill D.L., Fink W.J. (1991). Fluid replacement during prolonged exercise: effects of water, saline, or no fluid. Med Sci Sports Exerc.

[33] Walsh R.M., Noakes T.D., Hawley J.A., Dennis S.C. (1994). Impaired high-intensity cycling performance time at low levels of dehydration. Int J Sports Med.

[34] Murray S.R., Michael T.J., McClellan P.D. (1995). The influence of fluid replacement rate on heart rate and RPE during exercise in a hot, humid environment. J Strength Condit Res.

[35] Adams E.L., Casa D.J., Huggins R.A. (2019). Heat exposure and hypohydration exacerbate physiological strain during load carrying. J Strength Condit Res.

[36] Gava Pompermayer M., Rodrigues R., Manfredini Baroni B., de Oliveira Lupion R., Meyer F., Aurélio Vaz M. (2014). Rehydration during exercise in the heat reduces physiological strain index in healthy adults./Reidratação durante exercício no calor reduz o índice de esforço isiológico em adultos saudáveis. Braz J Kinanthropometry Hum Perform.

[37] James L.J., Moss J., Henry J., Papadopoulou C., Mears S.A. (2017). Hypohydration impairs endurance performance: a blinded study. Phys Rep.

[38] Wittbrodt M.T., Millard-Stafford M., Sherman R.A., Cheatham C.C. (2015). Fluid replacement attenuates physiological strain resulting from mild hypohydration without impacting cognitive performance. Int J Sport Nutr Exerc Metabol.

[39] Bardis C.N., Kavouras S.A., Adams J., Geladas N.D., Panagiotakos D.B., Sidossis L.S. (2017). Prescribed drinking leads to better cycling performance than ad libitum drinking. Med Sci Sports Exerc.

[40] Dugas J., Oosthuizen U., Tucker R., Noakes T. (2009). Rates of fluid ingestion alter pacing but not thermoregulatory responses during prolonged exercise in hot and humid conditions with appropriate convective cooling. Eur J Appl Physiol.

[41] McConell G.K., Stephens T.J., Canny B.J. (1999). Fluid ingestion does not influence intense 1-h exercise performance in a mild environment. Med Sci Sports Exerc.

[42] Muñoz C.X., Carney K.R., Schick M.K., Coburn J.W., Becker A.J., Judelson D.A. (2012). Effects of oral rehydration and external cooling on physiology, perception, and performance in hot, dry climates. Scand J Med Sci Sports.

[43] Perreault-Briere M., Beliveau J., Jeker D., Deshayes T.A., Duran A., Goulet E.D.B. (2019). Effect of thirst-driven fluid intake on 1 H cycling time-trial performance in trained endurance athletes. Sports.

[44] Ebert T.R., Martin D.T., Bullock N. (2007). Influence of hydration status on thermoregulation and cycling hill climbing. Med Sci Sports Exerc.

[45] Erkmen N., Taskin H., Kaplan T., Sanioglu A. (2010). Balance performance and recovery after exercise with water intake, sport drink intake and no fluid. J Exercise Sci Fitness.

[46] Backhouse S.H., Biddle S.J.H., Williams C. (2007). The influence of water ingestion during prolonged exercise on affect. Appetite.

[47] Robinson T.A., Hawley J.A., Palmer G.S. (1995). Water ingestion does not improve 1-h cycling performance in moderate ambient temperatures. Eur J Appl Physiol Occup Physiol.

[48] Lee M., Hammond K., Vasdev A. (2014). Self-selecting fluid intake while maintaining high carbohydrate availability does not impair half-marathon performance. Int J Sports Med.

[49] Lambert G.P., Lang J., Bull A. (2008). Fluid restriction during running increases GI permeability. Int J Sports Med.

[50] Berkulo M.A., Bol S., Levels K., Lamberts R.P., Daanen H.A., Noakes T.D. (2016). Ad-libitum drinking and performance during a 40-km cycling time trial in the heat. Eur J Sport Sci.

[51] Dion T., Savoie F.A., Asselin A., Gariepy C., Goulet E.D. (2013). Half-marathon running performance is not improved by a rate of fluid intake above that dictated by thirst sensation in trained distance runners. Eur J Appl Physiol.

[52] Ganio M.S., Wingo J.E., Carrolll C.E., Thomas M.K., Cureton K.J. (2006). Fluid ingestion attenuates the decline in VO2peak associated with cardiovascular drift. Med Sci Sports Exerc.

[53] Hillman A.R., Vince R.V., Taylor L., McNaughton L., Mitchell N., Siegler J. (2011). Exercise-induced dehydration with and without environmental heat stress results in increased oxidative stress. Appl Physiol Nutr Metabol.

[54] Hashimoto H., Ishijima T., Suzuki K., Higuchi M. (2016). The effect of the menstrual cycle and water ingestion on physiological responses during prolonged exercise at moderate intensity under hot conditions. J Sports Med Phys Fit.

[55] Muhamed A.M.C., Yusof H.A., Stannard S.R., Mündel T., Thompson M.W. (2019). The efficacy of ingesting water on thermoregulatory responses and running performance in a warm-humid condition. Front Physiol.

[56] Nuccio R.P., Barnes K.A., Carter J.M., Baker L.B. (2017). Fluid balance in team sport athletes and the effect of hypohydration on cognitive, technical, and physical performance. Sports Med.

[57] Maughan R.J., Watson P., Shirreffs S.M. (2015). Implications of active lifestyles and environmental factors for water needs and consequences of failure to meet those needs. Nutr Rev.

[58] Cheuvront S., Kenefick R. (2014). Dehydration: physiology, assessment, and performance effects. Compr Physiol.

[59] Goulet E.D., Hoffman M.D. (2019). Impact of ad libitum versus programmed drinking on endurance performance: a systematic review with meta-analysis. Sports Med.

[60] Adams W.M., Ferraro E.M., Huggins R.A., Casa D.J. (2014). Influence of body mass loss on changes in heart rate during exercise in the heat: a systematic review. J Strength Condit Res.

[61] Goulet E.D. (2011). Effect of exercise-induced dehydration on time-trial exercise performance: a meta-analysis. Br J Sports Med.

[62] Backhouse S.H., Bishop N.C., Biddle S.J., Williams C. (2005). Effect of carbohydrate and prolonged exercise on affect and perceived exertion. Med Sci Sports Exerc.

[63] Doherty M., Smith P. (2005). Effects of caffeine ingestion on rating of perceived exertion during and after exercise: a meta-analysis. Scand J Med Sci Sports.

[64] Jüni P., Witschi A., Bloch R., Egger M. (1999). The hazards of scoring the quality of clinical trials for meta-analysis. JAMA.

[65] Ali A., Moss C., Yoo M.J.Y., Wilkinson A., Breier B.H. (2017). Effect of mouth rinsing and ingestion of carbohydrate solutions on mood and perceptual responses during exercise. J Int Soc Sports Nutr.

[66] Costa R.J., Camões-Costa V., Snipe R.M., Dixon D., Russo I., Huschtscha Z. (2019). Impact of exercise-induced hypohydration on gastrointestinal integrity, function, symptoms, and systemic endotoxin and inflammatory profile. J Appl Physiol.

[67] Vallier J.M., Grego F., Basset F., Lepers R., Bernard T., Brisswalter J. (2005). Effect of fluid ingestion on neuromuscular function during prolonged cycling exercise. Br J Sports Med.

[68] Wingo J., Lafrenz A., Ganio M., Edwards G., Cureton K. (2005). Cardiovascular drift is related to reduced maximal oxygen uptake during heat stress. Med Sci Sports Exerc.

[69] Barwood M.J., Goodall S., Bateman J. (2018). The effect of hot and cold drinks on thermoregulation, perception, and performance: the role of the gut in thermoreception. Eur J Appl Physiol.

[70] Maughan R.J., Shirreffs S.M., Leiper J.B. (2007). Errors in the estimation of hydration status from changes in body mass. J Sports Sci.

[71] Baker L.B., Ungaro C.T., Sopeña B.C. (2018). Body map of regional vs. whole body sweating rate and sweat electrolyte concentrations in men and women during moderate exercise-heat stress. J Appl Physiol.

[72] Larose J., Boulay P., Sigal R.J., Wright H.E., Kenny G.P. (2013). Age-related decrements in heat dissipation during physical activity occur as early as the age of 40. PLoS One.

[73] Chapman C.L., Johnson B.D., Vargas N.T., Hostler D., Parker M.D., Schlader Z.J. (2020). Both hyperthermia and dehydration during physical work in the heat contribute to the risk of acute kidney injury. J Appl Physiol.

[74] Arney B.E., Glover R., Fusco A. (2019). Comparison of rating of perceived exertion scales during incremental and interval exercise. Kinesiology.

[75] Goulet E.D. (2013). Effect of exercise-induced dehydration on endurance performance: evaluating the impact of exercise protocols on outcomes using a meta-analytic procedure. Br J Sports Med.

[76] Peters J.L., Mengersen K.L. (2008). Meta-analysis of repeated measures study designs. J Eval Clin Pract.

[77] Lamb K.L., Eston R.G., Corns D. (1999). Reliability of ratings of perceived exertion during progressive treadmill exercise. Br J Sports Med.

[78] Garcin M., Wolff M., Bejma T. (2003). Reliability of rating scales of perceived exertion and heart rate during progressive and maximal constant load exercises till exhaustion in physical education students. Int J Sports Med.

[79] Borenstein M., Hedges L.V., Higgins J.P., Rothstein H.R. (2009).

[80] Higgins J., Altman D., Sterne A. (2011). Cochrane handbook for systematic reviews of interventions. Versiones.

[81] Evans G., Maughan R., Shirreffs S., James M.R. (2019). Lifestyle Medicine.

[82] Stover E.A., Petrie H.J., Passe D., Horswill C.A., Murray B., Wildman R. (2006). Urine specific gravity in exercisers prior to physical training. Appl Physiol Nutr Metabol.

[83] Merry T.L., Ainslie P.N., Walker R., Cotter J.D. (2008). Fitness alters fluid regulatory but not behavioural responses to hypohydrated exercise. Physiol Behav.

[84] Baker L.B., Munce T.A., Kenney W.L. (2005). Sex differences in voluntary fluid intake by older adults during exercise. Med Sci Sports Exerc.

[85] O'Neal E., Poulos S., Bishop P. (2012). Hydration profile and influence of beverage contents on fluid intake by women during outdoor recreational walking. Eur J Appl Physiol.

[86] Rhodes R.E., Kates A. (2015). Can the affective response to exercise predict future motives and physical activity behavior? A systematic review of published evidence. Ann Behav Med.

[87] Smirmaul Bde P. (2012). Sense of effort and other unpleasant sensations during exercise: clarifying concepts and mechanisms. Br J Sports Med.

[88] Cafarelli E. (1982). Peripheral contributions to the perception of effort. Med Sci Sports Exerc.

[89] McCloskey DI.Corollary discharges: motor commands and perception. In:Handbook of Physiology. The Nervous System. Motor Control, edited byBrooks VB. Bethesda, MD: Am. Physiol. Soc., 1981, sect. 1, vol. II, p.1415–1447

[90] Hampson D.B., St Clair Gibson A., Lambert M.I., Noakes T.D. (2001). The influence of sensory cues on the perception of exertion during exercise and central regulation of exercise performance. Sports Med.

[91] Amann M., Venturelli M., Ives S.J. (2013). Peripheral fatigue limits endurance exercise via a sensory feedback-mediated reduction in spinal motoneuronal output. J Appl Physiol.

[92] Noble B.J. (1996).

[93] Halperin I., Emanuel A. (2020). Rating of perceived effort: methodological concerns and future directions. Sports Med.

[94] Pereira G., Souza DMd, Reichert F.F., Smirmaul B.P.C. (2014). Evolution of perceived exertion concepts and mechanisms: a literature review. Revista Brasileira de Cineantropometria & Desempenho Humano..

[95] Pageaux B. (2016). Perception of effort in exercise science: definition, measurement and perspectives. Eur J Sport Sci.

[96] Straka H., Simmers J., Chagnaud B.P. (2018). A New perspective on predictive motor signaling. Curr Biol.

[97] Watson P., Head K., Pitiot A., Morris P., Maughan R.J. (2010). Effect of exercise and heat-induced hypohydration on brain volume. Med Sci Sports Exerc.

[98] Myers J., Atwood J.E., Sullivan M. (1987). Perceived exertion and gas exchange after calcium and beta-blockade in atrial fibrillation. J Appl Physiol.

[99] Davies C.T., Sargeant A.J. (1979). The effects of atropine and practolol on the perception of exertion during treadmill exercise. Ergonomics.

[100] Giersch G.E.W., Morrissey M.C., Butler C.R. (2021). Sex difference in initial thermoregulatory response to dehydrated exercise in the heat. Physiol. Rep..

