# Supplementary material for: Impact of dehydration on perceived exertion during endurance exercise: A systematic review with meta-analysis
Source: J Exerc Sci Fit. 2022 Apr 13;20(3):224–35. doi: 10.1016/j.jesf.2022.03.006 (PMC9093000; doi:10.1016/j.jesf.2022.03.006)
Supplement: Multimedia component 1 [file mmc1.docx]

**Impact of Exercise-induced Dehydration on Perceived Exertion During Endurance Exercise: A Systematic Review with Meta-analysis**

**Keywords and strategy used for the research of potential studies**

| **Hydration (S1)** | Hydrat* or Dehydrat* or Hypohydrat* or Euhydrat* or fluid or drink* | Title or abstract |
| --- | --- | --- |
| **Perceived exertion (S2)** | “Perceived exertion” or “Perceived effort” or “Rate of perceived exertion” or “Rating of perceived exertion” or “RPE” or percept* or exertion or effort | Title or abstract |
| **Exercise (S3)** | Exercise* or run* or cycl* or endurance or performance | Title or abstract |
| **Overall research** | S1 AND S2 AND S3 |  |

*Note: Research has been done on EBSCO with 4 different databases: SPORTDiscus, CINAHL, AMED and MEDLINE. The same research was also done using PubMed. Last search: February 16, 2022.*
